# Editorial: When the prefrontal cortex network goes wrong: the neuroinflammatory impact on behavioral disorders

**DOI:** 10.3389/fnbeh.2023.1345436

**Published:** 2024-01-08

**Authors:** Jonna M. Leyrer-Jackson, Mark D. Namba, Mark P. Thomas, M. Foster Olive

**Affiliations:** ^1^Department of Medical Education, School of Medicine, Creighton University, Phoenix, AZ, United States; ^2^Department of Pharmacology and Physiology, College of Medicine, Drexel University, Philadelphia, PA, United States; ^3^School of Biological Sciences, University of Northern Colorado, Greeley, CO, United States; ^4^Department of Psychology, Arizona State University, Tempe, AZ, United States

**Keywords:** prefrontal cortex, neuroinflammation, substance abuse, cytokine, immunotherapy

Prefrontal cortical areas are known to support goal-directed behaviors in humans, mediating a variety of functions that lead to behavioral flexibility/adaptability in response to continuously varying environmental demands. Normal prefrontal function relies on interconnectivity with subcortical brain structures, including the hippocampus, striatum and amygdala, as well as dopaminergic innervation and dopamine release from the ventral tegmental area. Dysregulation of prefrontal connectivity and function can contribute to a variety of psychopathologies and mood disorders, including schizophrenia, bipolar disorder, attentional deficits, anxiety disorders, post-traumatic stress disorder (PTSD), and compulsive drug-seeking. More recently, neuroinflammatory processes across multiple brain regions have been shown to alter neuronal function and regional connectivity, which in turn can contribute to a myriad of neurobehavioral impairments. While several studies suggest that neuroinflammatory processes may contribute to a number of etiologies, it remains of high importance to uncover the mechanism by which neuroinflammation within the prefrontal cortex and its interconnectivity can contribute to symptomology.

In this Research Topic entitled “*When the prefrontal cortex network goes wrong: the neuroinflammatory impact on behavioral disorders*” we, together with leaders in the field, have brought together the most recent findings and insights into neuroinflammation and its role in abnormal cognitive function and its underpinnings for various disease states. Our Research Topic includes 7 research articles that address several different brain regions and behaviors with an emphasis on neuroinflammation and neuroinflammatory markers. Three of the articles focus on substance abuse and inflammatory cytokines associated with drug/substance intake. One study highlights the effects of operant oral self-administration of alcohol on behavior and cytokine release in mice, finding that alcohol consumption increases stress levels measured by ultrasonic vocalization (Tonetto et al.). Tonetto et al. also report that alcohol consumption increases expression of the pro-inflammatory cytokine TNF-α within the prefrontal cortex, hippocampus, and striatum. In a study conducted in rats, Nagy et al. report findings that 3 weeks of binge-like intravenous intake of the synthetic cathinone 3,4-methylenedioxypyrovalerone (MDPV) followed by a 3-week period of forced abstinence induces elevated levels of the endothelial adhesion protein VCAM-1/CD106, which is thought to increase leukocyte trafficking across the blood-brain-barrier persisting into abstinence. In another study, Loftis et al. report that chronic methamphetamine exposure results in significantly reduced cognitive function and increases levels of macrophage inflammatory protein (MIP-2) expression within the frontal cortex in mice. Interestingly, post-exposure treatment with immunotherapies (DRmQ or Ibudilast) restores cognitive function and rescues MIP-2 expression in methamphetamine-treated mice (Loftis et al.). Taken together, these studies shed light on the neuroinflammatory underpinnings that may contribute to drug-seeking behavior and provide evidence that immunotherapies targeting neuroinflammatory substrates may prove beneficial for treating individuals with substance use disorders. While not the first studies to demonstrate the effects of substance use on neuroinflammatory mechanisms, these studies provide additional mechanisms by which substance use may alter cytokine-dependent cell signaling that may alter motivated behavior and impair cognitive function.

Further additions to this Research Topic focus on the contribution of mesocorticolimbic neuroimmune signaling to cognitive dysfunction within the context of cancer, pain, and mood disorders. For example, Demos-Davies et al. explore the underpinnings of cancer-related cognitive impairment following chemo- and radiation therapy, whereby mice treated with chemotherapy and radiation exhibit hippocampal dependent memory deficits due to enhanced activity of microglia and astrocytes. These findings were also associated with an increase in plasma expression of the cytokine interleukin-6 (IL-6), leading the authors to conclude that non-brain directed radiation and chemotherapy can increase gliosis and neurocognitive deficits in individuals receiving cancer treatment (Demos-Davis et al.). Furthermore, Tan et al. found that spinal cord injury (SCI) in rats increases inflammatory cytokine levels, including TNF-α, IL-6 and IL-1β, within the cortex, thalamus, midbrain, and medulla. SCI rats also exhibit enhanced microglial activation within the ventral tegmental area (VTA), which may lead to alterations in dopaminergic cell function and dopamine release at downstream targets such as the prefrontal cortex. Interestingly, treatment of SCI rats with transcranial direct current stimulation (tDCS) reduced proinflammatory cytokine levels in all brain regions explored and reduced microglial activation within the VTA, demonstrating promising therapeutic effects (Tan et al.). Similarly, a study by Wu et al. utilizes electroacupuncture (EA), a therapy utilized to treat depression, to alleviate depression-like symptoms and reduce inflammatory cytokine levels in mice following induction of a depression-like phenotype by lipopolysaccharide exposure. Wu et al. expand on these findings by demonstrating that EA reduces glutamate release within the prefrontal cortex and inhibits indoleamine 2,3-dioxygenase, an enzyme responsible for regulating the kynurenine pathway, which is hypothesized to reduce pain in individuals with SCI. Lastly, Shanazz et al. explore susceptibility and resiliency to PTSD in rats, finding that susceptibility to a PTSD-like phenotype is positively correlated with increased levels of IL-6 within the medial prefrontal cortex. Findings from this study suggest that neuroinflammation prior to trauma may render individuals more susceptible to stress-related neuropsychiatric disorders, such as PTSD.

The articles presented in this Research Topic provide interesting insights into neuroinflammation and the underlying etiologies of multiple disease states. Each study highlights important findings that contribute to our current understanding of, and reveal novel insights into, neuroimmune mechanisms that contribute to various disease states. Moreover, these studies provide evidence for the use of immunomodulatory therapies that may prove beneficial for treating a variety of disease states, including substance use disorders, depression, PTSD and SCIs. Together, these studies underscore the importance for additional exploration of neuroinflammation and its contribution to complex behaviors observed across various disease states.

## Author contributions

JL-J: Writing – original draft, Writing – review & editing. MN: Writing – review & editing. MT: Writing – review & editing. MO: Writing – review & editing.

